# Resiquimod induces a mixed Th1 and Th2 response via STAT1 and STAT3 signalling in chickens

**DOI:** 10.1016/j.bbrep.2025.101941

**Published:** 2025-02-04

**Authors:** Deepthi Kappala, Saravanan Ramakrishnan, Patel Nikunjkumar Prakashbhai, Abinaya Kaliappan, Prasad Thomas, Jörg Linde, Mithilesh Singh, Sohini Dey, Madhan Mohan Chellappa

**Affiliations:** a Immunology Section, ICAR - Indian Veterinary Research Institute, Bareilly, India; b Division of Bacteriology and Mycology, ICAR - Indian Veterinary Research Institute, Bareilly, India; c Institute of Bacterial Infections and Zoonoses, Friedrich-Loeffler-Institut, Jena, Germany; d Recombinant DNA Laboratory, Division of Veterinary Biotechnology, ICAR - Indian Veterinary Research Institute, Bareilly, India

**Keywords:** Resiquimod, Toll-like receptor signalling, Chicken, Th1 and Th2 immune responses, Transcriptomic profile

## Abstract

Resiquimod (R-848), a synthetic TLR7 agonist, modulates immune responses, primarily inducing Th1-biased immunity in mammals. In contrast, our previous studies revealed that R-848 stimulates both Th1 and Th2 responses in chickens. The current research investigates the molecular mechanisms underlying these immune responses in chickens. Pooled splenocytes harvested from chickens (n = 2/group) at 24 h post R-848 treatment were subjected to RNA sequencing and significant differentially expressed genes (DEGs) were identified compared to controls. Eight key genes associated with signal transduction (MAPK14, MAP3K8, PIK3CD, STAT1, STAT3, MAPK11, LRRK2, and GATA3) were validated via real-time PCR in chicken peripheral blood mononuclear cells (PBMCs) from six biological replicates. Pharmacological inhibitors or chloroquine were employed to elucidate the signalling pathways. SB202190, IC-87114, and fludarabine phosphate suppressed the R-848-induced expression of MAPK14, PIK3CD, and STAT1, respectively, while chloroquine decreased STAT3 expression. Intriguingly, chloroquine treatment enhanced the R-848-mediated expression of MAPK11, LRRK2, and GATA3. These results align with transcriptomic findings and highlight the upregulation of STAT1 and STAT3 as potential contributors to the induction of Th1 and Th2 immune responses by R-848 in chickens. These insights provide a foundation for optimizing R-848 as an immunomodulatory agent in avian species.

## Highlights

• Signalling molecules involved in resiquimod (R-848) mediated immune responses in chickens are investigated.
• The DEGs were validated by real-time PCR.
• Transcriptome profile showed association of eight significant DEGs with R-848 signal transduction.
• STAT1 and STAT3 upregulation by R-848 have contributed to Th1 and Th2 immune responses, respectively in chickens.

## 1 Introduction

Toll-like receptors (TLRs) play a critical role in the early detection of pathogens, bridging innate and adaptive immunity. Chickens express ten TLRs (TLR1A, TLR1B, TLR2A, TLR2B, TLR3, TLR4, TLR5, TLR7, TLR15, and TLR21), each recognizing distinct pathogen-associated molecular patterns [[1], [2], [3], [4]]. Among these, TLR7, located on endosomal membranes, detects single-stranded RNA (ssRNA) and initiates signalling cascades via the myeloid differentiation primary-response protein 88 (MyD88)-dependent pathway. This process results in the activation of NF-κB and MAPK pathways, leading to the production of pro-inflammatory cytokines and subsequent immune responses [[5], [6], [7], [8]].

Resiquimod (R-848), a synthetic TLR7 agonist, has been extensively studied for its immunomodulatory effects in mammals, where it primarily induces Th1 responses characterized by IFN-γ and IL-12 production [9,10]. For instance, R-848 treatment mediated the suppression of established allergic asthma in mice by decreasing Th2 cytokines (IL-4, IL-5, IL-13) while increasing the production of Th1 cytokines (IL-12 and IFN-γ) [11]. Similarly, intranasal administration of R-848 suppressed allergic airway disease in humans [12]. However, our previous research demonstrated that R-848 induces both Th1 and Th2 responses in chickens. For example, the intramuscular administration of R-848 up-regulated the expression of IFN-β, IFN-γ, IL-1β, IL-4 and iNOS transcripts in broiler, White Leghorn and Kadaknath breed of chickens [13]. Similarly, co-administration of R-848 with inactivated Newcastle disease virus (NDV) vaccine enhanced both humoral and cellular immune responses and also afforded complete protection against virulent NDV challenge [14]. In addition, R-848 also showed prophylactic effect against vvIBDV challenge in White Leghorn birds [15]. Recently, R-848 adjuvanted live or inactivated avian infectious bronchitis virus (IBV) vaccine showed an increase in both systemic and mucosal immune responses [16]. Despite its demonstrated efficacy, the mechanisms underlying R-848-mediated immune modulation in chickens remain poorly understood. While studies have elucidated the signalling pathways activated by TLR7 agonists in mammals, little is known about the specific signalling molecules and pathways involved in avian species. Addressing this knowledge gap is crucial for optimizing the use of R-848 as an adjuvant in poultry. This study hypothesizes that R-848 activates distinct signalling molecules within the TLR7 pathway, contributing to the observed mixed Th1/Th2 immune response in chickens. To test this hypothesis, we performed RNA sequencing (RNA-seq) on splenocytes from R-848-treated chickens to identify differentially expressed genes (DEGs) and validated these findings using pharmacological inhibitors and chloroquine. By elucidating the signalling pathways involved, this research aims to provide a mechanistic basis for R-848-mediated immune responses, with potential applications in enhancing vaccine efficacy and boosting immune resilience in poultry which helps in improving the overall health and productivity of poultry.

## 2 Methods

### 2.1 Experimental birds, study design and ethics

Specific pathogen-free (SPF) eggs of White Leghorn breed were purchased from Venky's India Pvt. Limited, Pune, India. Eggs were hatched at the Central Avian Research Institute, Izatnagar. Standard management practices were followed for the maintenance of birds. Ad libitum feed and water were provided to the birds throughout the experiment. The total experiment was officially approved by the Institute Animal Ethical Committee (IAEC), ICAR-IVRI, Bareilly, India [File No. F. 26-1/2015-16/JD(R) dated 18^th^ May, 2017 and F.26-2/2019/JD(R) dated 6th Jan 2020]. All the animal experiments were performed in accordance with the IVRI-IAEC guidelines and regulations. Further, animal studies were reported in compliance with the ARRIVE guidelines.

Four weeks old unvaccinated birds (n = 2) of White Leghorn breed of either sex, were administered with R-848 through intramuscular route at a dose rate of 50 μg per chicken [14]. Birds (n = 2) of either sex injected with phosphate buffered saline (PBS) served as control. Two birds per group were employed as per the previous reports [[17], [18], [19]] and due to IAEC approval. After 24 h of R-848 and PBS administration, birds were humanely sacrificed and spleen samples were pooled within each group in diethyl pyrocarbonate (DEPC, Amresco, USA) treated eppendorf tubes containing Trizol (Amresco, USA). Pooling of samples for RNA-seq experiments enhances the identification of differentially expressed genes (DEGs) [20].

### 2.2 RNA extraction and sequencing

The samples were processed for RNA extraction and sequencing. Briefly, total RNA was isolated from the chicken spleen samples using Direct-zol RNA Miniprep Kit. RNA concentration and purity were assessed by Nanodrop spectrophotometry at A260/A280. The quality control (QC) passed RNA samples were subjected to cDNA library construction using TruSeq stranded mRNA Library Prep Kit (Illumina, USA) as per the manufacturer's guidelines. Then, the cDNA libraries were sequenced using an IlluminaNextSeq 500 platform to generate paired-end reads of 150 bp in length (Eurofins Pvt Ltd, India).

### 2.3 Transcriptome analysis and identification of differentially expressed genes

Analysis of transcriptomic (RNA-seq) data was carried out using Geo2rnaseq R pipeline [21]. Within the pipeline, quality control and quality trimming were carried out using FastQC (https://www.bioinformatics.babraham.ac.uk/projects/fastqc/) and Trimmomatic [22], respectively. Read mapping of trimmed reads was carried out with the reference genome GRCg6a (Accession number GCA_000002315.5) for chicken (*Gallus galllus*) using TopHat2 [23]. Read counting was performed using feature counts [24]. Finally, differentially expressed genes (DEGs) were calculated using baySeq [25]. Genes with cutoff value of false discovery rate (FDR) < 0.5 and log_2_ fold change (FC) > 0 were considered as DEGs. The RNA-seq data was submitted to the NCBI Gene Expression Omnibus (GEO) database with experiment series accession number GSE180434.

### 2.4 Functional annotation and protein-protein interaction (PPI) network predictions

Functional annotation of genes was performed using g:Profiler [26]. The Gene Ontology (GO) terms associated with the DEGs were assigned for the categories such as biological processes, molecular function and cellular component. Eight DEGs associated with signal transduction pathways were subjected to further analysis. For protein-protein interaction (PPI) network predictions, the String tool was applied (https://string-db.org/) [27] with a medium confidence score (>0.4) [28].

### 2.5 Validation experiments using RT-qPCR

A total of eight genes identified as DEG associated with signal transduction were further validated by quantitative real time PCR in the chicken PBMCs. Chloroquine and specific inhibitors against selected signalling molecules were used to determine the pathway involved in R-848 induced TLR7 stimulation. Chloroquine, as an endosomal acidification blocker, inhibits the entire TLR7 signaling pathway by preventing the acidification required for ligand recognition and receptor activation.

#### 2.5.1 Stimulation of chicken PBMCs with TLR7 agonist

Blood was collected from two weeks old SPF birds (n = 6) in syringes containing heparin (20 IU/mL) and was transferred to 15 mL centrifuge tubes containing an equal volume of Ficoll-Histopaque 1.077 (Sigma, MO, USA) to separate PBMCs aseptically by density gradient centrifugation. The cells were washed twice with phosphate-buffered saline (PBS, pH 7.2) and re-suspended in RPMI-1640 complete medium containing 2 % fetal bovine serum and 100 IU/mL penicillin and 100 μg/mL streptomycin. The cell viability was determined by the trypan blue dye exclusion method and the cell count was adjusted to 2 × 10^6^ cells/mL. The PBMCs were stimulated with R-848 (2 μg/mL) [29] and incubated at 40 °C with 5 % CO_2_. The untreated cells served as control.

#### 2.5.2 Inhibition experiment using chloroquine and specific inhibitor against selected signalling molecules

The PBMCs from two weeks old SPF birds (n = 6) were pre-treated with chloroquine at 100 μM [30] and incubated for 30 min at 40^o^C with 5 % CO_2_. After 30 min of incubation, the cells were stimulated with R-848 and harvested at 12 and 24 h post-stimulation to determine the relative expression of signalling molecules (MAP3K8, STAT3, GATA3, MAPK11 and LRRK2) as compared to the cells stimulated with R-848 in the absence of inhibitor.

Specific inhibitors against signalling molecules MAPK14 (SB202190), PIK3CD (IC-87114) and STAT1 (Fludarabine phosphate) were resuspended according to manufacturer's instructions. The following concentrations were used: SB202190 10 μM [31], IC-87114 25 μM [32] and Fludarabine phosphate 25 μM [33]. The PBMCs from 2-weeks old SPF birds (n = 6) were pre-treated with or without indicated concentrations of SB202190 for 1 h at 40^o^C with 5 % CO_2_. Subsequently, the cells were stimulated with R-848 and further incubated for 3, 6, 12 h. Similarly, the indicated concentration of fludarabine phosphate was used for treatment of the PBMCs followed by stimulation with R-848 and further incubation for 12 and 24 h. The PIK3CD inhibitor IC-87114 was added to the chicken PBMCs at the time of R-848 addition and incubated for 12 and 24 h [33]. The harvested cells at different time intervals were used to determine the relative expression of selected signalling genes by RT-qPCR.

#### 2.5.3 RNA isolation, cDNA synthesis and real time PCR

Total RNA was isolated from the harvested cells based on previous literature [29] and was stored at −80^o^ C until further use. The cDNA synthesis was carried out using RevertAid First Strand cDNA Synthesis Kit (Thermo Scientific, USA) using random hexamer primer following manufacturer protocols. Briefly, total RNA isolated from PBMCs was reverse transcribed to cDNA. Primer mix containing 2 μg total RNA and 1 μL of random hexamer (Thermo Scientific, USA) was incubated at 65 °C for 5 min and kept in ice for 5 min. Then the master mix containing 5 × reaction buffer, Ribolock RNase inhibitor, 10 mM dNTP mix, and RevertAid reverse transcriptase were added to the primer mix. This mixture was incubated at 25 °C for 10 min followed by 50 °C for 50 min for cDNA synthesis and the reaction was terminated by heating at 85 °C for 5 min. The cDNA product was stored at −20 °C until further use.

The relative expression of MAPK14, MAP3K8, PIK3CD, STAT1, STAT3, MAPK11 LRRK2, and GATA3 transcripts was analyzed by real-time PCR using a QuantiFast SYBR Green qPCR kit (Qiagen, CA, USA) on a CFX 96 Real-Time System (Bio-Rad, CA, USA). In addition, the expression levels of mRNA of IL-1β, IFN-β, IFN-γ and IL-4 were quantified using real-time PCR from inhibition experiment using specific inhibitor against MAPK14, PIK3CD and STAT1 molecules. The primers for GAPDH, MAPK14, MAP3K8, PIK3CD, STAT1, STAT3, MAPK11 LRRK2, and GATA3 were designed using primer3 software (http://bioinfo.ut.ee/primer3/) and synthesized from M/S. Integrated DNA Technologies, Iowa, USA. The sequence of the primers and their annealing temperatures are given in Table 1. The house keeping gene GAPDH was used to normalize the expression level of the target genes. The real-time PCR mixture consisted of 2 μL cDNA, 10 μL QuantiFast SYBR Green Master Mix, primers (0.5 μL each, 10 pmol concentration), and nuclease-free water to a volume of 20 μL. Real time PCR was performed using the following program: first cycle at 95 °C for 5 min, followed by 39 cycles each of 94 °C for 30 s, annealing for 45 s, 70 °C for 45 s and a final cycle of 94 °C for 30 s. The final step was to obtain a melting curve for determining amplification specificity. Each sample was run in duplicate on the same plate. The difference in cycle threshold (ΔCt) values for the target and GAPDH gene was calculated. The ΔCt of the untreated cells served as a calibrator to calculate the relative fold change of the target genes in R-848 plus inhibitor groups using the 2^−ΔΔCt^ method [34].

**Table 1:** Primer sequences used in real time PCR reaction.

Target Genes	Primer sequence (5′-3′)	Annealing temperature (^o^C)	Product size (bp)	Accession number
GAPDH	F:5′-GTGGTGCTAAGCGTGTTATCATC-3′	60	269	NM_204305.2
	R: 5′-GGCAGCACCTCTGCCATC3′			
MAPK14	F: 5′-GGACTTGCTAGAGAAGATGTTGGT-3′	58	109	XM_419263.7
	R: 5′-TGGTTCGTCATCTGGGTCATG-3′			
MAP3K8	F: 5′-TCTGTCATGGAGAAGCTGGAGA-3′	61	97	XM_025148361.3
	R: 5′-AGTGGAGAAAGTCGAGCCCT-3′			
PIK3CD	F: 5′-TGGGCAGCTTGGATGGAGTA-3′	59	84	NM_001012696.2
	R: 5′-GGGTGTGTGCAAGAGTCCAG-3′			
STAT1	F: 5′-CACCACAGAGCACACACAGA-3′	59	104	NM_001012914.2
	R: 5′-CAAGACAGGCATTGGGTGGG-3′			
STAT3	F: 5′-ACTACAGACTCGGCAGCAGA-3′	60	85	NM_001398325.1
	R: 5′-CCGGTGTTGGACAATTGGGT-3′			
MAPK11	F: 5′-GAACAAGACGGTGTGGGAGG-3′	62	90	NM_001006227.2
	R: 5′-TCATAAGCTGAACACACGGAGC-3′			
LRRK2	F: 5′-TGCACCCGTGTCCTTGAAAG-3′	60	113	NM_001287193.3
	R: 5′-TGGCATTGAACAGCTCCAGT-3′			
GATA3	F: 5′-CGTTTACCCGCCTGCTTCTT-3′	61	119	NM_001008444.2
	R: 5′-CAGGAGTGGAGATGGACGGA-3′			
IL-1β	F: 5′-GGATTCTGAGCACACCACAGT-3′	60	272	NM_204524.2
	R: 5′-TCTGGTTGATGTCGAAGATGTC-3′			
IFN-β	F: 5′-GCTCACCTCAGCATCAACAA-3′	60	187	NM_001024836.2
	R: 5′-GGGTGTTGAGACGTTTGGAT-3′			
IFN-γ	F: 5′-TGAGCCAGATTGTTTCGATG-3′	60	152	NM_205149.2
	R: 5′-CTTGGCCAGGTCCATGATA-3′			
IL-4	F: 5′-GTGCCCACGCTGTGCTTAC-3′	60	82	NM_001007079.2
	R: 5′-AGGAAACCTCTCCCTGGATGTC-3′			

### 2.6 Statistical analysis

All the validation experiments using real-time PCR were performed with six biological replicates per group. Statistical analysis was conducted using two-way ANOVA to determine the significance of differences between treatment groups and time points. To address multiple comparisons, *post hoc* (Tukey's) test was applied following ANOVA to identify specific group differences that contributed to significant results. P-values <0.05 were considered statistically significant. Results are presented as mean ± SE (standard error). Graphical representation of relative fold expression was created using GraphPad Prism 8.0.1 (San Diego, CA, USA).

## 3 Results

### 3.1 Gene expression profile of chicken spleen following R-848 administration

In the current study, we aimed for the identification of significant DEGs in the chicken spleen following R-848 administration in comparison to PBS control group. On analysis, 5885 significantly DEGs (including up-regulated and down-regulated genes) were identified in the R-848 treated birds. Further, the functional annotation of these DEGs were carried out using g:Profiler to infer significant GO terms (biological process, molecular function and cellular component) associated. The significant biological processes and molecular functions identified among DEG are detailed in g:Profiler results GSE180434.

Among the DEGs, 3125 genes were upregulated, and 2760 were downregulated. Notably, eight genes associated with signal transduction pathways—MAPK14, MAP3K8, PIK3CD, STAT1, STAT3, MAPK11, LRRK2, and GATA3—were selected for further analysis due to their potential roles in immune modulation. The fold change values for these genes are summarized in Table 2.

**Table 2:** Eight selected DEGs and their functions in immune response.

DEGs	Log2 fold change^a^	FDR	Functions	References
MAPK14	0.62	0.45	Involve in the expression of pro-inflammatory immune response genes such as IL-6 and TNF-α	[35]
MAP3K8	0.38	5.13E-10	MAP3K8 is an upstream regulator of MAPK and NF-kB activation	[36]
PIK3CD	0.27	0.42	Involve in the activation of B cell functions and immunoglobulin secretion. Further, it is also involved in the expression of IL-6, iNOS, TNF-α and type I IFNs	[32,37]
STAT1	0.53	0.11	STAT1 is important for the differentiation of Th1 cells by increasing the expression of T-bet	[38]
STAT3	0.39	0.45	STAT3 negatively regulates immunostimulatory effects. STAT3 also co-operates with STAT6 in development of Th2 cells	[39]
MAPK11	−0.81	0	MAPK11 is expressed on inflammatory and stress stimuli	[40]
LRRK2	−0.44	0	LRRK2 has a role in innate immunity and is the downstream target of IKK which is important in the expression of IL-1β, TNF-α and IL-6	[41,42]
GATA3	−0.50	0	GATA3 differentiates the naïve T cells to Th2 phenotype	[43]

a The cut off value for log_2_FC and FDR are >0 and <0.5, respectively.

**Fig. 1:**
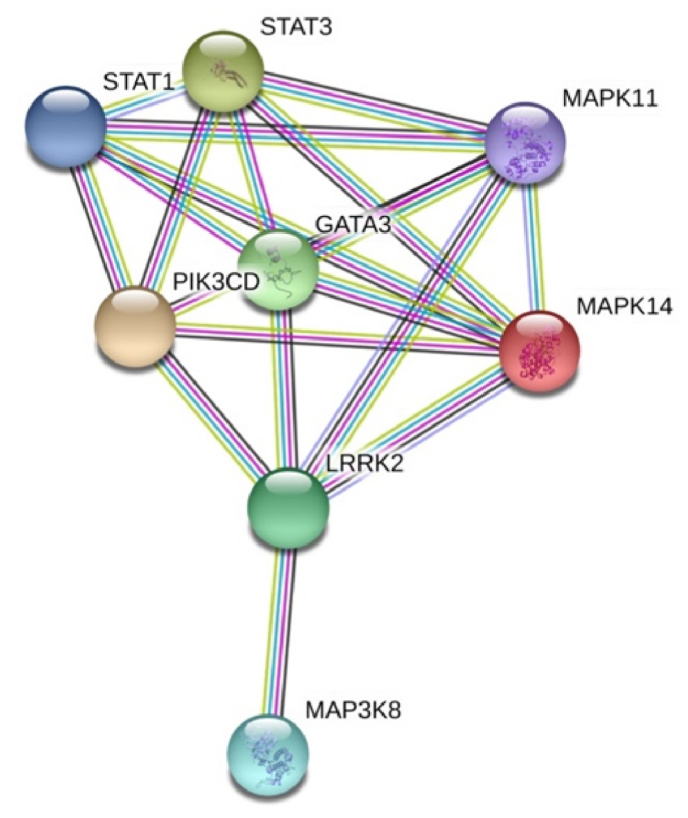
Protein-protein interaction network generated from eight selected genes with 8 nodes and 19 edges. Each line colour indicates the type of interaction evidence. Functional associations determined from database, experiments, textmining and co-expression are indicated by blue, pink, green and black, respectively.

To construct a representative protein-protein interaction network, eight DEGs (MAPK14, MAP3K8, PIK3CD, STAT1, STAT3, MAPK11, LRRK2 and GATA3) involved in biological processes associated with signal transduction pathways were considered. The protein-protein interaction network among the eight DEGs resulted in 8 nodes and 19 edges (Fig. 1).

### 3.2 Effect of MAPK14, PIK3CD, STAT1 inhibitors on R-848 mediated expression of immune response genes in the chicken PBMCs

**Fig. 2:**
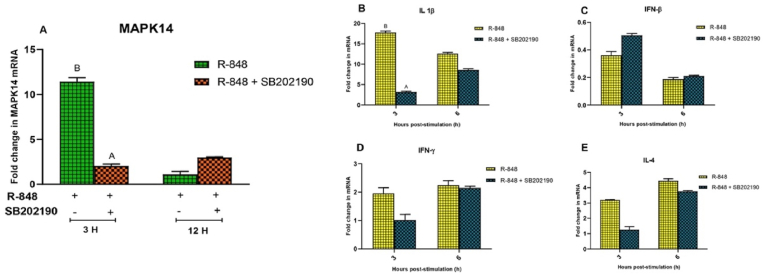
Effect of specific inhibitor targeting MAPK14 gene on the R-848 mediated expression of IL-1β, IFN-β, IFN-γ and IL-4 transcripts in the chicken peripheral blood mononuclear cells (PBMCs). **(2A-E)** Relative expression of MAPK14 mRNA and immune response gene transcripts in chicken PBMCs stimulated with R-848 (2 μg/mL) or R-848 (2 μg/mL) plus MAPK14 specific inhibitor (SB202190; 10 μM) for 12 h. Two way ANOVA was carried out to estimate the effect of stimulation. Alpha error was minimised by calculating the adjusted P value (P < 0.05). Different superscripts above bars (mean ± SE) indicate significant effects of R-848 and R-848 plus SB202190 in the chicken PBMCs at respective time interval.

SB202190, specific inhibitor of MAPK14, significantly decreased the expression of MAPK14 (2.044 ± 0.202 folds) at 3 h post-stimulation with R-848 in the chicken PBMCs as compared to the R-848 alone (11.417 ± 0.45 folds) stimulated cells (Fig. 2A). However, there was no significant difference in the expression of MAPK14 mRNA level at 12 h post-stimulation in either of the two groups. This transient downregulation highlights MAPK14's role in the early phase of immune responses. Consequently, pro-inflammatory cytokines regulated by MAPK14 were measured at 3 and 6 h, as later time points would not capture these early effects. Inhibition of MAPK14 using SB202190 significantly decreased (P < 0.05) the expression of IL-1β mRNA (3.19 ± 0.22 folds) at 3 h post-stimulation with R-848 as compared to the R-848 alone (17.785 ± 0.391 folds) stimulated chicken PBMCs. However, no difference was observed in the expression level of IL-1β transcript at 6 h post-stimulation between the groups (Fig. 2B). SB202190 did not show any statistically significant effect on the R-848 mediated expression of IFN-β, IFN-γ and IL-4 mRNA in the chicken PBMCs (Fig. 2B–E). While the upregulation of MAPK14 was modest (1.5-fold), its role in activating downstream pro-inflammatory cytokines such as IL-1β and IFN-β suggests that even subtle changes in expression may initiate significant immune responses. MAPK14's involvement in the p38 MAPK pathway is critical for amplifying immune signalling, particularly in the context of pathogen recognition. Therefore, this increase, although subtle, could play a key role in priming the immune system for an effective response.

**Fig. 3:**
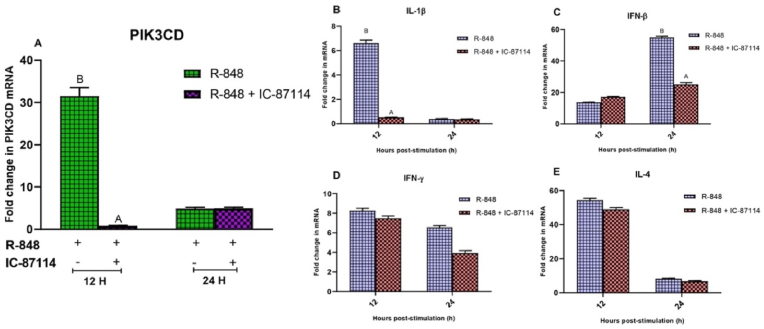
Effect of specific inhibitor targeting PIK3CD gene on the R-848 mediated expression of IL-1β, IFN-β, IFN-γ and IL-4 transcripts in the chicken peripheral blood mononuclear cells (PBMCs). **(3A-E)** Relative expression of PIK3CD mRNA and immune response gene transcripts in the chicken PBMCs stimulated with R-848 (2 μg/mL) or R-848 (2 μg/mL) plus PIK3CD specific inhibitor (IC-87114; 25 μM) for 24 h. Two way ANOVA was carried out to estimate the effect of stimulation. Alpha error was minimised by calculating the adjusted P value (P < 0.05). Different superscripts above bars (mean ± SE) indicate significant effects of R-848 and R-848 plus IC-87114 in the chicken PBMCs at respective time interval.

The chicken PBMCs stimulated with R-848 showed increased expression of PIK3CD transcripts at 12 h (31.544 ± 1.97 folds) and 24 h (4.874 ± 0.32 folds) intervals. Pre-incubation of chicken PBMCs with IC-87114 significantly decreased (0.822 ± 0.06 folds) the expression of PIK3CD at 12 h post-stimulation with R-848 as compared to the R-848 alone (31.544 ± 1.97 folds) stimulated cells. IC-87114 showed no change in the PIK3CD transcript level (4.953 ± 0.28 folds) at 24 h post-stimulation with R-848 as compared to R-848 alone (4.874 ± 0.32 folds) stimulated chicken PBMCs (Fig. 3A). Further, PIK3CD inhibitor (IC-87114) significantly turned down (P < 0.05) the expression of IL-1β mRNA (0.514 ± 0.02 folds) at 12 h post-stimulation with R-848 as compared to the R-848 alone (6.608 ± 0.26 folds) stimulated cells (Fig. 3B). Similarly, the expression of IFN-β mRNA was significantly (P < 0.05) reduced (25.119 ± 1.19 folds) after using IC-87114 at 24 h post-stimulation with R-848 as compared to the R-848 alone (54.939 ± 0.77 folds) stimulated chicken PBMCs (Fig. 3C). However, the expression level of IFN-γ and IL-4 mRNA was not affected by IC-87114 at 12 and 24 h intervals in the chicken PBMCs (Fig. 3D and E).

**Fig. 4:**
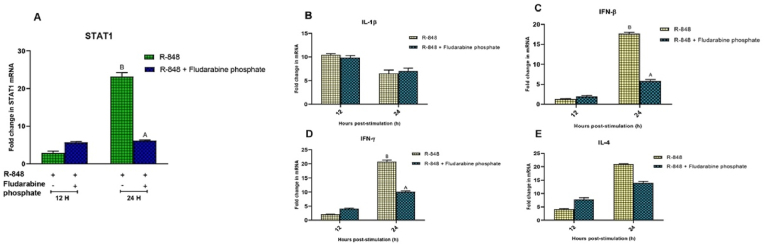
Effect of specific inhibitor targeting STAT1 gene on the R-848 mediated expression of IL-1β, IFN-β, IFN-γ and IL-4 transcripts in the chicken peripheral blood mononuclear cells (PBMCs). **(4A-E)** Relative expression of STAT1 mRNA in the chicken PBMCs stimulated with R-848 (2 μg/mL) or R-848 (2 μg/mL) plus Fludarabine phosphate; 25 μM) for 24 h. Two way ANOVA was carried out to estimate the effect of stimulation. Alpha error was minimised by calculating the adjusted P value (P < 0.05). Different superscripts above bars (mean ± SE) indicate significant effects of R-848 and R-848 plus Fludarabine phosphate in the chicken PBMCs at respective time interval.

R-848 induced STAT1 mRNA expression at 12 h (2.927 ± 0.44 folds) and 24 h (23.187 ± 1.06 folds) post-stimulation in the chicken PBMCs. Peak expression of transcript was observed at 24 h interval. Fludarabine phosphate decreased the expression of STAT1 transcript (6.173 ± 0.17 folds) at 24 h post-stimulation with R-848 in the chicken PBMCs as compared to the R-848 alone (23.187 ± 1.06 folds) stimulated cells. However, fludarabine phosphate did not show any inhibitory effect on STAT1 expression at 12 h post-stimulation with R-848 (Fig. 4A). In addition, fludarabine phosphate significantly diminished (P < 0.05) the expression of IFN-β (5.827 ± 0.37 folds) and IFN-γ (10.095 ± 0.3 folds) transcripts at 24 h post-stimulation with R-848 as compared to R-848 alone (IFN-β: 17.695 ± 0.33 and IFN-γ: 20.761 ± 0.54 folds, respectively) stimulation in the chicken PBMCs (Fig. 4C and D). STAT1 inhibitor showed no significant changes in the expression of IL-1β and IL-4 transcripts at 12 and 24 h intervals in the chicken PBMCs (Fig. 4B–E).

### 3.3 Effect of chloroquine on the R-848 mediated expression of MAP3K8, STAT3, MAPK11, LRRK2 and GATA3 in the chicken PBMCs

**Fig. 5:**
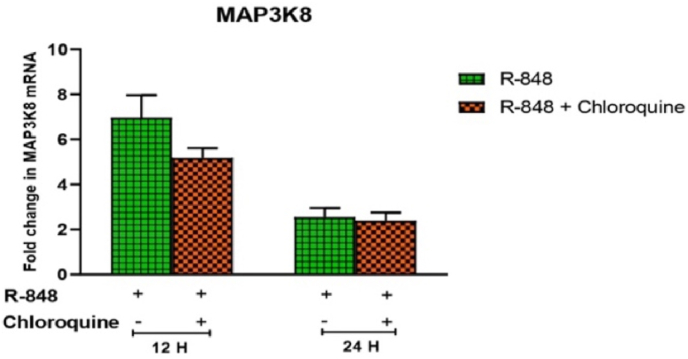
Relative expression of MAP3K8 mRNA in the chicken peripheral blood mononuclear cells (PBMCs) stimulated with R-848 (2 μg/mL) or R-848 (2 μg/mL) plus chloroquine (100 μM) for 24 h. Two way ANOVA was carried out to estimate the effect of stimulation. Alpha error was minimised by calculating the adjusted P value (P < 0.05). Different superscripts above bars (mean ± SE) indicate significant effects of R-848 and R-848 plus chloroquine in the chicken PBMCs at respective time interval.

R-848 stimulated chicken PBMCs showed the expression of MAP3K8 transcript at 12 h (6.963 ± 0.99 folds) and 24 h (2.553 ± 0.41 folds) post-stimulation. Chloroquine decreased the R-848 mediated MAP3K8 transcript level (5.182 ± 0.42 folds) at 12 h post-stimulation numerically, which was not statistically significant (Fig. 5).

**Fig. 6:**
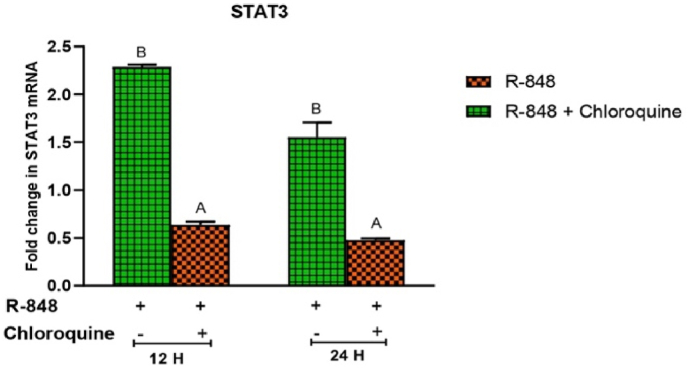
Relative expression of STAT3 mRNA in the chicken peripheral blood mononuclear cells (PBMCs) stimulated with R-848 (2 μg/mL) or R-848 (2 μg/mL) plus chloroquine (100 μM) for 24 h. Two way ANOVA was carried out to estimate the effect of stimulation. Alpha error was minimised by calculating the adjusted P value (P < 0.05). Different superscripts above bars (mean ± SE) indicate significant effects of R-848 and R-848 plus chloroquine in the chicken PBMCs at respective time interval.

R-848 induced the expression of STAT3 mRNA at 12 h (2.289 ± 0.02) and 24 h (1.551 ± 0.16) post-stimulation in the chicken PBMCs. Chloroquine treated cells showed significantly (P < 0.05) reduced R-848 mediated STAT3 mRNA expression at 12 h (0.638 ± 0.03 folds) and 24 h (0.477 ± 0.01 folds) interval as compared to the R-848 alone (2.289 ± 0.02 and 1.551 ± 0.16 folds respectively) stimulated cells (Fig. 6).

**Fig. 7:**
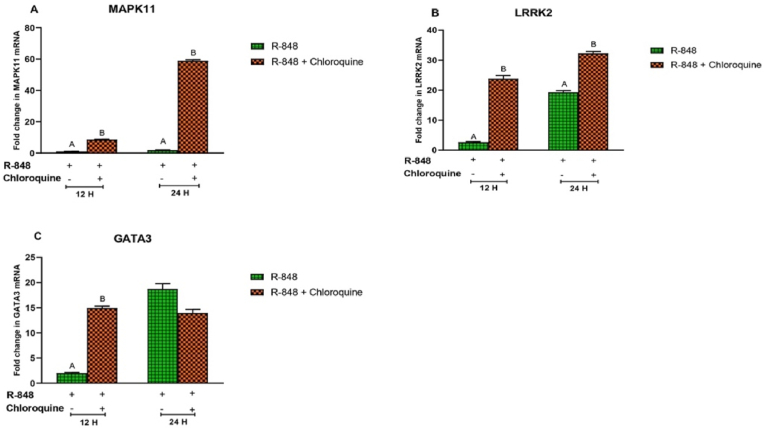
Relative expression of MAPK11 (A), LRRK2 (B), and GATA3 (C) mRNA in the chicken peripheral blood mononuclear cells (PBMCs) stimulated with R-848 (2 μg/mL) or R-848 (2 μg/mL) plus chloroquine (100 μM) for 24 h. Two way ANOVA was carried out to estimate the effect of stimulation. Alpha error was minimised by calculating the adjusted P value (P < 0.05). Different superscripts above bars (mean ± SE) indicate significant effects of R-848 and R-848 plus chloroquine in the chicken PBMCs at respective time interval.

Pre-incubation of chicken PBMCs with chloroquine significantly (P < 0.05) increased the R-848 mediated expression of MAPK11 and LRRK2 transcripts at 12 h (8.689 ± 0.24 and 23.801 ± 1.08 folds, respectively) and 24 h (58.91 ± 0.66 and 32.257 ± 0.62 folds, respectively) post-stimulation as compared to the R-848 alone stimulated cells. Peak expression level of MAPK11 (Fig. 7A) and LRRK2 (Fig. 7B) transcripts was observed at 24 h interval in chloroquine incubated chicken PBMCs.

Chloroquine significantly (P < 0.05) increased the R-848 mediated GATA3 mRNA expression (14.936 ± 0.37 folds) at 12 h post-stimulation as compared to the R-848 alone (1.982 ± 0.16) stimulated cells. However, chloroquine showed no inhibitory effect on the R-848 mediated expression of GATA3 mRNA at 24 h interval (Fig. 7C).

**Fig. 8:**
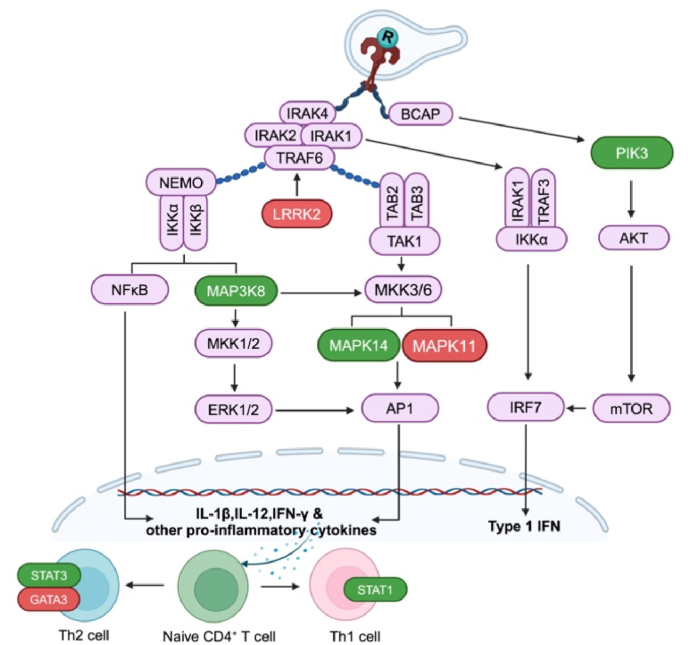
R-848 signalling cascade in chickens. Green and red colored boxes indicates the upregulated and downregulated genes, respectively in this study. AKT (Protein kinase B); AP1 (Activator protein 1); IRF7 (interferon regulatory factor 7); BCAP (B-cell adaptor protein); ERK1/2 (Extracellular signal-regulated kinase); GATA3 (GATA binding protein 3). IKKα/β (Inhibitor of nuclear factor-κB (IκB) kinase alpha/beta); IRAK1/2/4 (Interleukin 1 receptor associated kinase 1/2/4); MAP3K8 (mitogen-activated protein kinase kinase kinase 8); MAPK11/14 (mitogen-activated protein kinase 11/14); MKK1/2/3/6 (Mitogen-activated protein kinase kinase 1/2/3/6); mTOR (mammalian target of rapamycin); NEMO (Nuclear factor-kappa B Essential Modulator); NF-κB (nuclear factor-kappa B); TAB2/3 (TGF-beta activated kinase 2/3); PIK3 (Phosphatidylinositol-4,5-bisphosphate 3-kinase); R (R-848); STAT1/2 (signal transducer and activator of transcription); TAK1 (Transforming growth factor-β (TGF-β)-activated kinase 1); TRAF3/6 (Tumor necrosis factor receptor associated factor 3/6).The figure was made using bioRender tool.

Additionally, the eight DEGs associated with the R-848 signalling cascade in chickens are illustrated in Fig. 8.

## 4 Discussion

The present study investigates the molecular mechanisms underlying R-848-mediated immune responses in chickens, focusing on the signaling pathways that drive balanced Th1 and Th2 responses. While previous research from our lab established R-848 as a potent activator of Th1 and Th2 immunity and a candidate vaccine adjuvant, the signalling molecules responsible for these effects were not fully elucidated. Using transcriptomic profiling and validation experiments, we identified key genes involved in R-848-induced immune modulation, providing insights into their roles in cytokine regulation and immune pathway activation.

### 4.1 MAPK signalling pathway

The p38MAPK family members consist of p38α (MAPK14), p38β (MAPK11), p38γ (MAPK12) and p38δ (MAPK13) and are activated through classical MAPK cascade among which p38α is considered to be a central regulator of inflammatory responses [40]. p38β is 75 % identical to p38α and these two isoforms are best studied in inflammatory conditions [44]. Our findings highlight the role of MAPK14 (p38α) in initiating pro-inflammatory immune responses. Transcriptomic and RT-qPCR analyses revealed upregulation of MAPK14 following R-848 stimulation, consistent with its established role in mediating cytokine production, such as IL-1β and TNF-α [35]. Pharmacological inhibition using SB202190 demonstrated a significant reduction in MAPK14 and IL-1β expression, emphasizing its involvement in early immune signaling. In concordance with our study, stimulation of human carcinoma cells and gingival keratinocytes using *Porphyromonas gingivalis* membrane fractions containing TLR agonists showed upregulation of MAPK14 gene [45]. MAPK14 inhibitor reduced accumulation of IL-6 in the supernatant of granulosa cells suggesting the involvement of MAPK14 in IL-6 production from the Pam or LPS stimulated cells [46]. Conversely, MAPK11 (p38β), another isoform of the p38 MAPK family, was downregulated, reflecting a differential regulation of p38 isoforms in chickens. This is in consistent with a study wherein infection of goat PBMCs with non-cytopathic BVDV-2 strain (positive sense ss-RNA virus) differentially downregulated MAPK11 in transcriptomic profile [47].

### 4.2 NF-κB and MAP3K8 pathways

NF-κB is a central regulator of immune responses, activated through canonical and non-canonical pathways [48,49]. MAP3K8, also known as Tpl2 kinase, serves as a critical kinase that bridges the MAPK and NF-κB signalling pathways [36]. In resting cells, MAP3K8 forms a complex with NF-κB inhibitory protein NF-κB1 p105, blocking its access to substrate MEK (MKK1/2). Upon TLR stimulation, NF-κB1 p105 is phosphorylated by the IKK complex, leading to its ubiquitination and degradation by the proteasome [50]. This process releases MAP3K8 to activate MKK1, MKK2, MKK3, and MKK6, driving downstream signalling cascades. For example, LPS-stimulated macrophages rely on MAP3K8-mediated activation of MKK3/6, which depends on NF-κB1 p105 phosphorylation [51]. MAP3K8 also catalyzes TNF-mediated activation of the MKK3/6-p38α axis in macrophages stimulated by TLR2, TLR4, and TLR7, directly activating the IKKβ-Tpl2-ERK inflammatory axis [52]. In this study, transcriptomic analysis demonstrated increased MAP3K8 expression in R-848-treated chickens, suggesting its critical role in activating MAPK and NF-κB pathways. Validation through RT-qPCR confirmed numerical upregulation of MAP3K8 transcripts in R-848-stimulated chicken PBMCs, which is consistent with the transcriptomic data.

### 4.3 PIK3CD and STAT signalling pathway

PIK3CD, a catalytic subunit of PI3K, plays a pivotal role in immune cell signalling. After activation by TLR agonist, PI3K recruits and phosphorylates protein kinase B (Akt) [53]. Glycogen synthase kinase3-β (GSK3-β) is a downstream target of Akt. Phosphorylation of GSK3-β results in its inactivation and regulates transcription factors responsible for pro- or anti-inflammatory cytokine production [54]. For instance, inhibition of GSK3-β in TLR2, TLR4, TLR5 and TLR9 stimulated human monocytes decreased IL-6, TNF and IL-12 production while increase in IL-10 secretion. Further, stimulation of TLR7 and TLR9 on plasmacytoid dendritic cells also drive PI3K/Akt pathway which had positive role in type I IFN production [55]. In line with these findings, the upregulation of PIK3CD in transcriptomic and RT-qPCR analyses in our study suggests its involvement in R-848-mediated pro-inflammatory and antiviral responses. Notably, IC-87114, a PIK3CD inhibitor, significantly reduced the expression of IL-1β and IFN-β, confirming its role in cytokine production. Temporal analysis revealed peak PIK3CD expression at 12 h, aligning with its regulation of type I IFN responses.

STAT1 transcription factor is primarily activated by IFN-γ through JAK/STAT pathway and primes Th1 differentiation [38]. Stimulation of RAW264.7 cells with TLR7 agonist, loxoribine or R-848 differentially up-regulated STAT1, an innate gene responsible for viral defense in transcriptomic profile [56]. Similarly, administration of ducklings with lethal duck hepatitis A virus 3 (ss-RNA virus) induced STAT1 which promoted the production of IFN-α and IFN-β in transcriptomic profile [57]. Consequently, the successful immune response against infectious pathogen should be nullified by the anti-inflammatory environment to prevent detrimental tissue damage. In contrast to STAT1, IL-10 signalling brings phosphorylation and activation of STAT3 through JAK1 kinase which abrogates the inflammatory response by transcriptionally repressing pro-inflammatory cytokine genes such as IL-1β, IL-6, IL-12, and TNF-α. STAT3 negatively regulates the lethal type I IFN signalling pathway by inhibiting expression of IRF7, IRF9, STAT1 and STAT2 [58]. STAT3 also co-operates with STAT6 in the development of Th2 cells [39]. In concordance to these findings, STAT1 and STAT3, transcription factors critical for Th1 and Th2 differentiation, were differentially regulated in this study. STAT1 upregulation was associated with increased IFN-γ expression, establishing a Th1-biased immune environment. Interestingly, chloroquine treatment reduced STAT3 expression, suggesting its modulation by endosomal signalling pathways. The interplay between STAT1 and STAT3 highlights their complementary roles in balancing immune responses.

### 4.4 GATA3 and LRRK2 regulation

GATA3, a master regulator of Th2 differentiation [43], was downregulated following R-848 stimulation, as confirmed by transcriptomic and RT-qPCR data. This downregulation may result from R-848-mediated STAT1 activation, which antagonizes GATA3 expression. Similarly, LRRK2, a kinase involved in innate immunity and a downstream target of IKK [41], was downregulated in R-848-treated PBMCs, suggesting alternate regulatory mechanisms compensating for its reduced expression or species-specific differences in signalling mechanisms. These findings provide new insights into the modulation of Th2-associated pathways by R-848 in chickens.

Finally, our results confirm the role of MAPK14 in pro-inflammatory signalling through the p38 MAPK pathway. The upregulation of MAPK14 and its downstream impact on cytokines such as IL-1β and IFN-β align with its established role in mammalian systems, underscoring its conserved function across species. Similarly, the observed upregulation of STAT1 supports its well-documented role in promoting Th1 immune responses, as evidenced by increased IFN-γ expression in R-848-stimulated PBMCs.

By critically analyzing these findings, our study bridges existing knowledge gaps and provides a nuanced understanding of the roles of MAPK14, STAT1, and STAT3 in chicken immune responses. These insights not only confirm previously established roles but also highlight novel aspects of immune signalling in avian systems, laying the groundwork for future investigations into their biological and practical implications.

Though we have used real-time PCR assays for validation of the RNA-seq experimental data, the present study has limitation of using small sample size for RNA-seq data generation. However, earlier workers also used two birds per group for RNA-seq experiments [[17], [18], [19]]. Pooling of spleen samples was followed in this study for enhancing the detection of DEGs while reducing inter-individual variability [20]. Further, RNA-seq experiments have some inherent disadvantages such as representation of long transcripts by more reads than shorter transcripts. Further studies are needed incorporating Western blot analysis of some important signalling molecules such as STAT1, STAT3, MAP3K8 and MAPK14, which could provide additional confirmation of R-848 mediated immune responses in the chicken. In this study, we have validated the eight selected DEGs. Validation experiments involving more number of DEGs are further suggested.

## 5 Conclusion

Our findings provide critical insights into the transcriptomic changes induced by R-848 in chickens, linking these molecular alterations to functional immune responses. Upregulation of key signalling genes such as STAT1 and STAT3 correlates with enhanced expression of cytokine genes IFN-γ and IL-4, respectively in chickens. These cytokines play pivotal roles in shaping Th1 and Th2 responses, respectively, contributing to a balanced immune profile. Importantly, the induction of IL-1β and IFN-β suggests a robust pro-inflammatory and antiviral response, which is essential for effective pathogen resistance.

Further, the results also emphasize the potential of R-848 as a vaccine adjuvant in poultry. By inducing both Th1 and Th2 responses, R-848 can enhance both humoral and cellular immunity, as evidenced by its ability to upregulate genes associated with cytokine production and immune activation. These transcriptomic changes align with previous reports of R-848 improving vaccine efficacy against pathogens such as NDV and IBV.

In conclusion, this study not only elucidates the molecular mechanisms underlying R-848-mediated immune modulation but also highlights its translational potential as an immunomodulatory agent in poultry. By connecting transcriptomic changes to functional immune responses, we lay the groundwork for future research aimed at optimizing vaccine strategies and improving disease resilience in chickens.

### 5.1 Future directions

While this study identifies critical signalling pathways, further research is needed to validate these findings at the protein level and assess their functional implications in vivo. Additionally, exploring the cross-regulation between MAPK, NF-κB, and PI3K pathways will provide deeper insights into the molecular basis of R-848-mediated immunity.

## CRediT authorship contribution statement

**Deepthi Kappala:** Writing – review & editing, Writing – original draft, Methodology, Formal analysis, Data curation, Conceptualization. **Saravanan Ramakrishnan:** Writing – review & editing, Writing – original draft, Validation, Supervision, Software, Resources, Methodology, Investigation, Funding acquisition, Formal analysis, Data curation, Conceptualization. **Patel Nikunjkumar Prakashbhai:** Writing – original draft, Methodology, Data curation. **Abinaya Kaliappan:** Writing – review & editing, Writing – original draft, Software, Formal analysis, Data curation. **Prasad Thomas:** Writing – review & editing, Writing – original draft, Software, Formal analysis, Data curation. **Jörg Linde:** Writing – review & editing, Software, Formal analysis. **Mithilesh Singh:** Writing – review & editing, Resources, Data curation. **Sohini Dey:** Writing – review & editing, Resources. **Madhan Mohan Chellappa:** Writing – review & editing, Resources.

## Data availability

The datasets generated and analyzed during the current study are available in the NCBI Gene Expression Omnibus (GEO) repository, with experiment series accession number GSE180434. Further inquiries can be directed to corresponding author.

## Declaration of generative AI and AI-assisted technologies in the writing process

During the preparation of this work the author(s) didn't use any AI tools.

## Funding

This research did not receive any specific grant from funding agencies in the public, commercial, or not-for-profit sectors.

## Declaration of competing interest

The authors declare that they have no known competing financial interests or personal relationships that could have appeared to influence the work reported in this paper.
