# Supplementary material for: Resiquimod induces a mixed Th1 and Th2 response via STAT1 and STAT3 signalling in chickens
Source: Biochem Biophys Rep. 2025 Feb 4;41:101941. doi: 10.1016/j.bbrep.2025.101941 (PMC11847045; doi:10.1016/j.bbrep.2025.101941)
Supplement: Multimedia component 1 [file mmc1.docx]

Supplementary Material

**Supplementary Fig. 1.** Bar diagram displaying the summary of differentially expressed genes (DEGs) in chicken spleen from R-848 treated birds

**Supplementary Fig. 2.** Top enriched GO terms including categories of biological process, cellular component, molecular function (coloured green, blue and red, respectively) with p-value <0.05


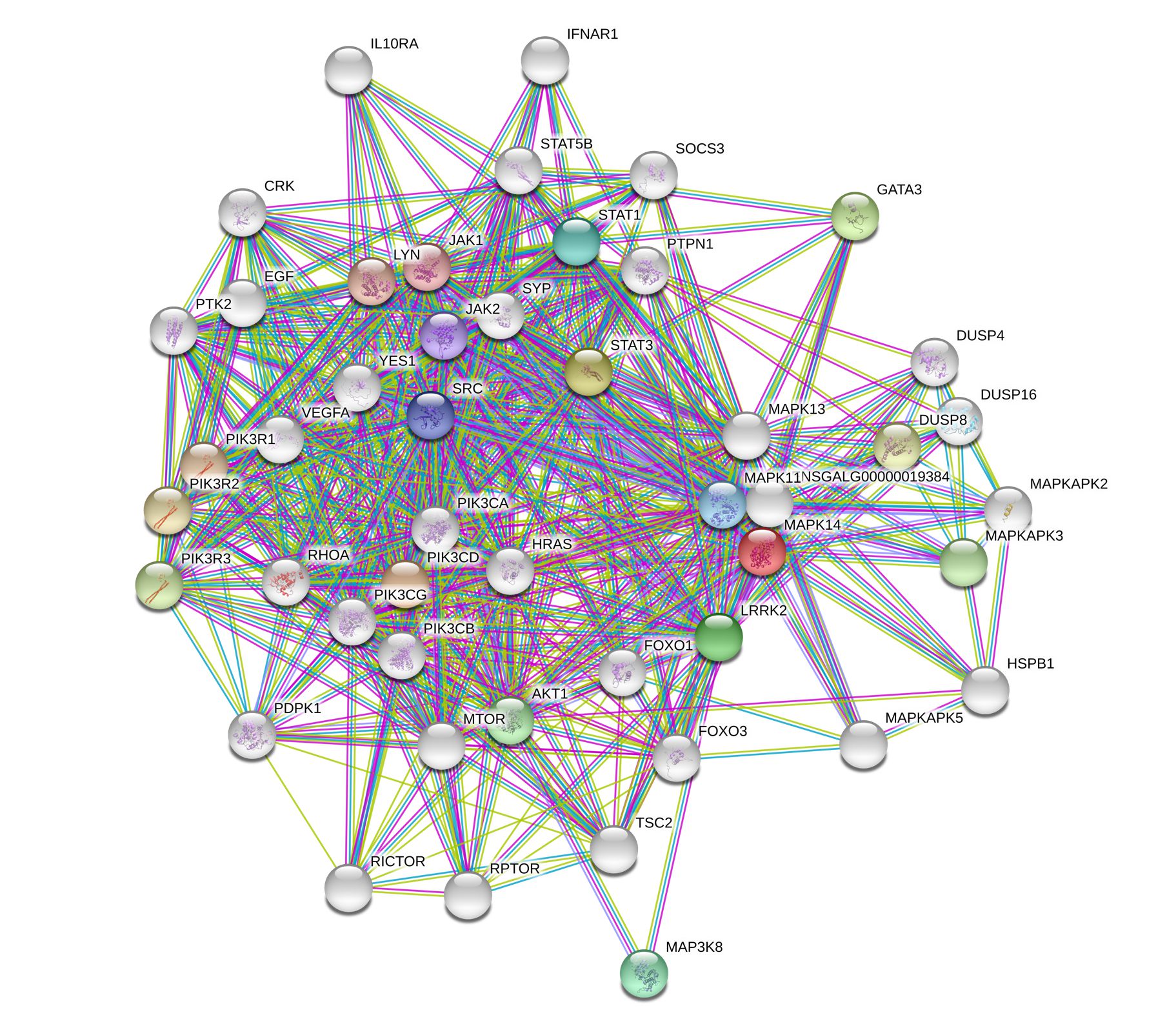


**Supplementary Fig. 3.** Large protein-protein interaction network with predicted functional partners


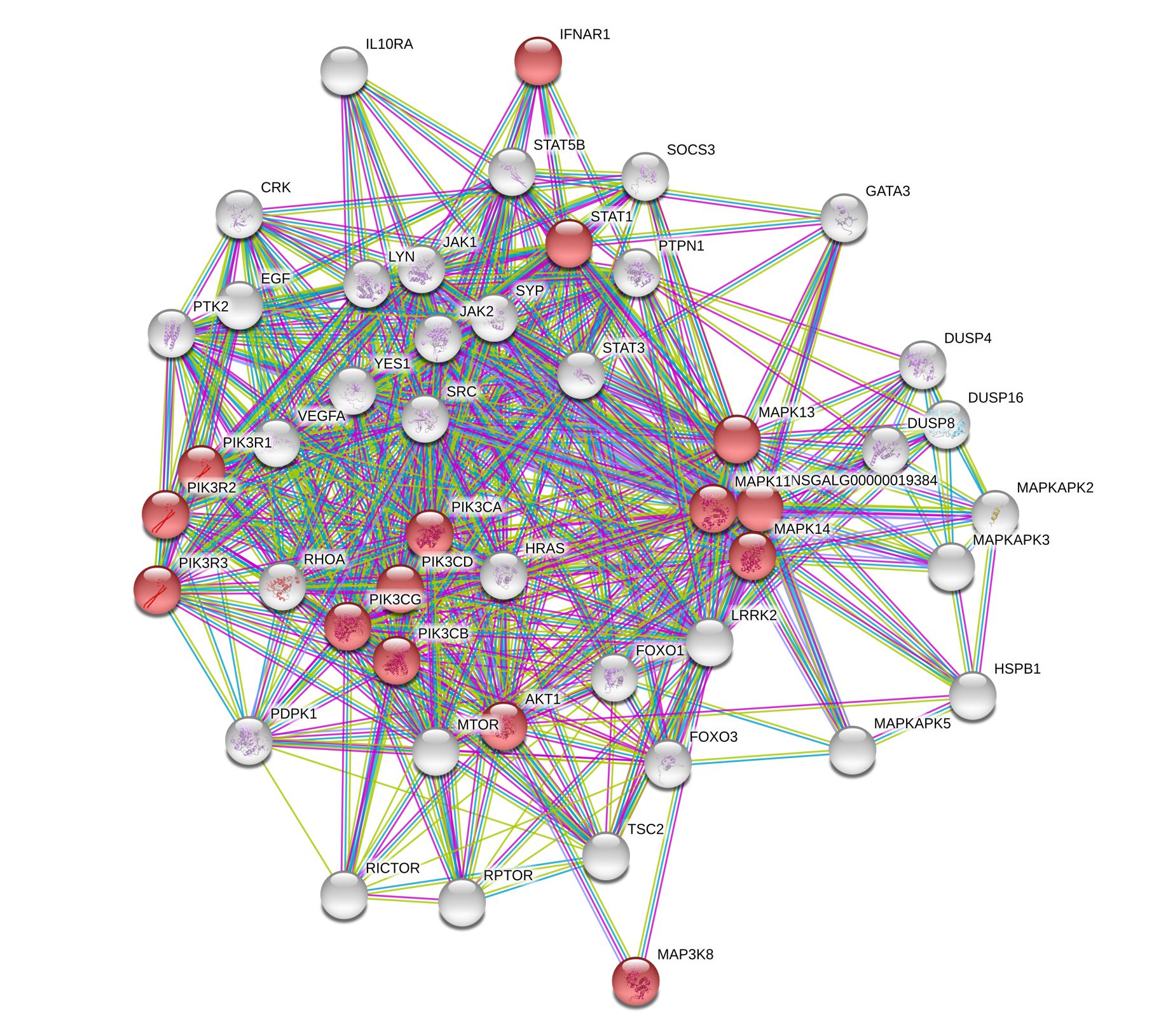


**Supplementary Fig. 4.** Proteins involved in Toll-like receptor signalling pathway indicated by red colour
